# Effects of elevated CO_2_ on fine root biomass are reduced by aridity but enhanced by soil nitrogen: A global assessment

**DOI:** 10.1038/s41598-017-15728-4

**Published:** 2017-11-10

**Authors:** Juan Piñeiro, Raúl Ochoa-Hueso, Manuel Delgado-Baquerizo, Silvan Dobrick, Peter B. Reich, Elise Pendall, Sally A. Power

**Affiliations:** 1 Hawkesbury Institute for the Environment, Western Sydney University, Locked Bag 1797, Penrith, New South Wales 2751 Australia; 2 Department of Forest Resources, University of Minnesota, St. Paul, Minnesota USA

## Abstract

Plant roots play a crucial role in regulating key ecosystem processes such as carbon (C) sequestration and nutrient solubilisation. Elevated (e)CO_2_ is expected to alter the biomass of fine, coarse and total roots to meet increased demand for other resources such as water and nitrogen (N), however, the magnitude and direction of observed changes vary considerably between ecosystems. Here, we assessed how climate and soil properties mediate root responses to eCO_2_ by comparing 24 field-based CO_2_ experiments across the globe including a wide range of ecosystem types. We calculated response ratios (i.e. effect size) and used structural equation modelling (SEM) to achieve a system-level understanding of how aridity, mean annual temperature and total soil nitrogen simultaneously drive the response of total, coarse and fine root biomass to eCO_2_. Models indicated that increasing aridity limits the positive response of fine and total root biomass to eCO_2_, and that fine (but not coarse or total) root responses to eCO_2_ are positively related to soil total N. Our results provide evidence that consideration of factors such as aridity and soil N status is crucial for predicting plant and ecosystem-scale responses to future changes in atmospheric CO_2_ concentrations, and thus feedbacks to climate change.

## Introduction

Plant roots play a major role in regulating important ecosystem functions such as nutrient cycling, C sequestration and plant productivity^1,2^. Coarse roots (>2 mm) can account for up to 40% of total biomass in terrestrial ecosystems and represent a large fraction of the more stable plant C pool^3^. The proportion of fine roots (<2 mm) varies between ecosystems, from 5–10% of standing total root biomass in forests to more than 50% in grasslands^4–6^. Even in ecosystems where fine roots represent a small proportion of standing root biomass, they can transfer up to 65% of the C-fixed annually by the canopy to the soil^4,7^. Increases in atmospheric CO_2_ concentrations (eCO_2_) have the potential to alter the standing biomass of fine and coarse roots differently. While fine root responses are generally considered in the context of altered demand for soil resources, the response of coarse/total root biomass to eCO_2_ can be seen as a proxy for changes in whole plant long-lived biomass^8^. For example, after eight years of CO_2_ exposure at the Duke-Free Air CO_2_ Enrichment (FACE) experiment, plots receiving increased CO_2_ had 17% greater coarse root biomass compared to ambient plots^9^, which was similar to the 19% CO_2_-enhancement of basal area during the same period^10^.

Field experiments tend to show enhanced belowground biomass under eCO_2_^9,11,12^. However, the direction and magnitude of these responses vary across different study sites. For instance, during 11 years of CO_2_ treatment in a scrub-oak shrubland in Florida, eCO_2_ enhanced fine root biomass only after natural disturbances (i.e. fire and hurricane)^13^, an effect that was attributed to increased resource availability, including nutrients, water and space. Similarly, four years of eCO_2_ did not result in any enhancement of fine root biomass in a late successional alpine treeline ecosystem in Switzerland^14^. Interestingly, some field experiments even report negative eCO_2_ effects on fine root production^15,16^, suggesting that the response of root biomass to eCO_2_ may be driven by multiple interactions with other environmental drivers such as climate and soil properties.

Soil resource availability, including soil N and water, may partly explain contrasting root biomass responses to eCO_2_^17–19^. For example, while eCO_2_ in conjunction with N fertilization has generally been reported to increase coarse and total root biomass^11^, the magnitude of eCO_2_ stimulation of fine root biomass can either increase^20^ or decrease compared to unfertilized plots exposed to eCO_2_^6,21^. Water limitation is generally believed to amplify aboveground plant growth responses to eCO_2_^17,22^, however, much less is known about the importance of water availability in controlling the response of belowground biomass to eCO_2_, with contrasting results reported in the recent literature^19,23,24^. For example, there was no interaction between CO_2_ and drought treatments for root biomass in a temperate grassland ecosystem^24^, whereas others found a positive effect of eCO_2_ on total root biomass only under well-watered conditions^23^. Such contrasting effects under similar treatment combinations may be due to intrinsic differences in climate and soil properties among ecosystems. However, to date, we lack a system-level understanding of the major environmental factors, including climate and nutrient availability, regulating the response of root biomass to eCO_2_ at the global scale. This information is critical for predicting ecosystem-level responses to global change and to properly integrate biosphere-atmosphere feedbacks into Earth System Models^25^.

**Figure 1:**
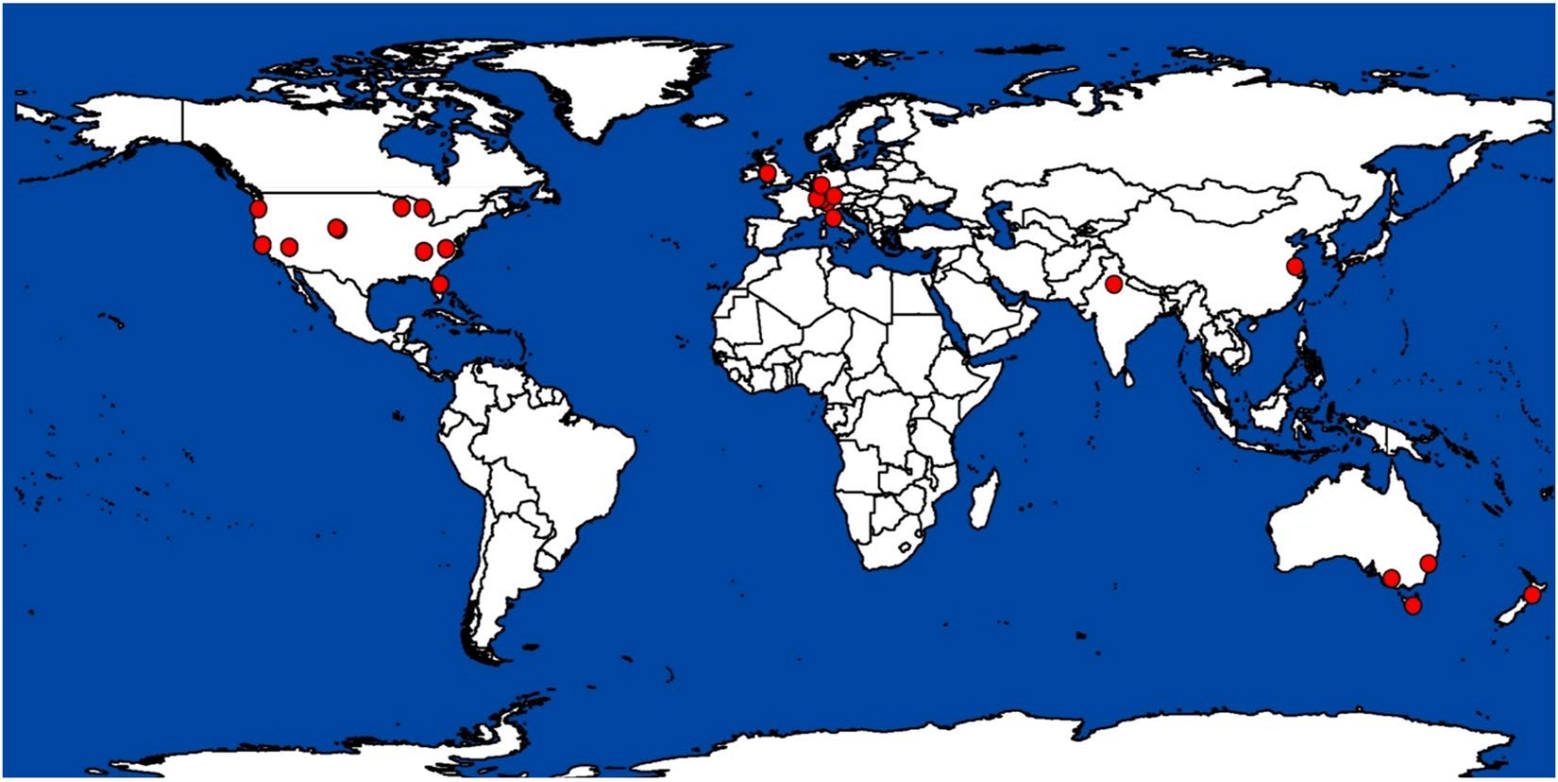
Location of the study sites included in the analysis. This map was created using ArcGIS Desktop 10 (Redlands, CA). http://www.esri.com/.

We used structural equation modelling (SEM) to evaluate how environmental factors regulate root biomass responses to eCO_2_ using data from 24 field-based experiments across multiple climate and vegetation types (Fig. 1). Previous analyses have examined overall eCO_2_ effects on root pools, and differences between ecosystem types, experimental facilities, plant functional groups and N fertilization^11,12,26^. In this study, however, we evaluated the role of climate (aridity and temperature) and soil conditions (soil pH, total soil C and N content) in regulating the magnitude of the responses of total, coarse and fine root biomass to eCO_2_ (as measured using the lnRR-response ratio^27^). Therefore, we do not focus on main CO_2_ effects on belowground biomass pools, which have been presented elsewhere^11,12^.

Given that increased plant C uptake under eCO_2_ may increase the demand for other resources like water and soil nutrients, the magnitude of eCO_2_ effects on fine root biomass has been predicted to be more pronounced under water and/or N limitation^8,28^. Herein, we hypothesize that the magnitude of fine root biomass responses to eCO_2_ (i.e. the response ratio) will be greater in ecosystems with lower levels of soil water (i.e. higher site aridity) or N content as a functional adaptation of plants to meet resource demand. In addition, multiple limitation theory suggests that the magnitude of the CO_2_ fertilization effect on overall plant growth should be limited by low availability of soil resources such as water and N, and some studies support this hypothesis^18,19^. Therefore, we hypothesize that low water and soil N content will decrease the magnitude of total root biomass responses to eCO_2_. We also posit that the response of fine roots to eCO_2_ will be more sensitive to water and nutrient limitation than that of coarse roots because coarse root biomass represents long-lived, structural tissues that are less responsive to rapid environmental change.

## Results

A total of 27 experiments complied with our selection criteria. However, two of these were conducted in peatlands and one in an alpine ecosystem, reporting soil characteristics from a thick organic layer (up to 20–30 cm.). The disproportionate (more than 10-times higher) C and N contents of the organic layer on these ecosystems compared to the remaining sites (mineral soils) included in the database, and the unique characteristics of C, nutrient and decomposition dynamics in these ecosystems^29^, led to their exclusion from the analysis. Of the remaining 24 experimental sites, 20, 7 and 15 experiments reported total, coarse and fine root biomass respectively (Fig. 1); seven studies reported responses for both coarse and fine root pools. Since some experiments included multiple species or time points, the database comprised 41 case studies for fine root biomass, 20 for coarse root biomass and 37 for fine root biomass. Overall, 18 experiments were conducted in temperate, 3 in arid and 3 in continental climates (Fig. 1). Of these 24 experiments, 11 were conducted in grasslands, 10 in forest/shrubland ecosystems and 3 in agricultural systems.

**Figure 2:**
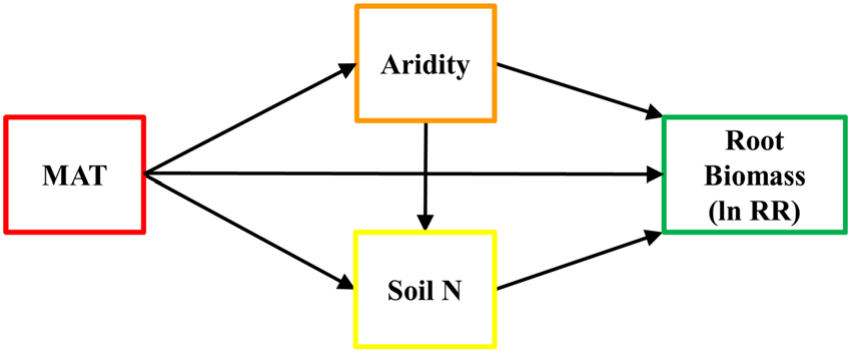
*A priori* structural equation model depicting the direct and indirect influences of MAT, Aridity and soil N on root biomass responses to eCO_2_ (lnRR). Boxes indicate measured variables entered in the model.

**Figure 3:**
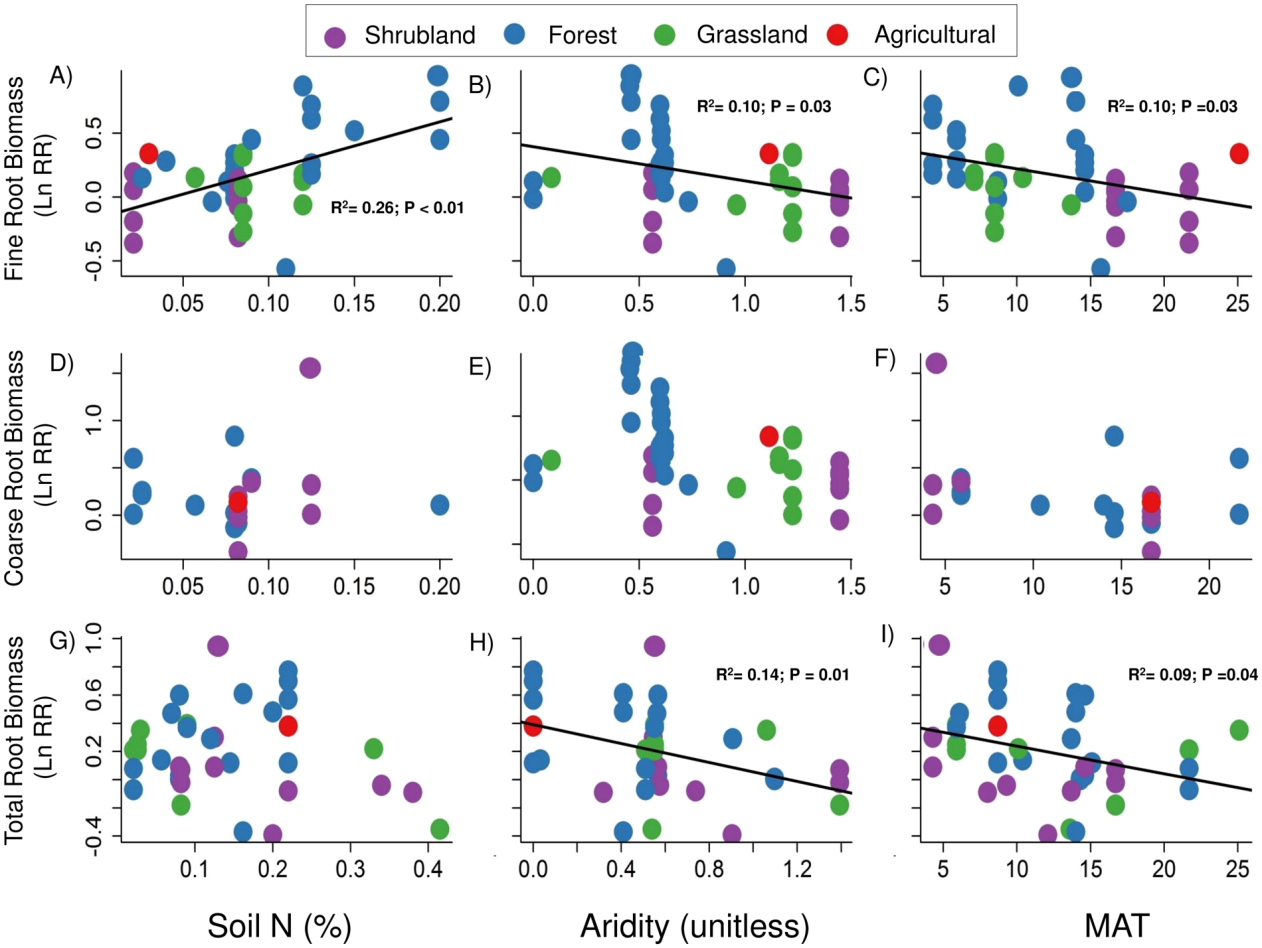
Relationships between cumulative fine, coarse and total root biomass responses to CO_2_ and site-level explanatory variables used in this study. The solid lines represent the fitted linear correlations. Aridity was calculated as [AI in this dataset – AI in each site] so high values of aridity represent places with low water availability.

Our *a priori* model (Fig. 2) incorporated the predictor variables that showed stronger correlations with root responses to eCO_2_ (Table 1; Fig. 3). Correlation analysis indicated that fine root responses to eCO_2_ were smaller at low levels of total soil N and high levels of aridity and MAT (Fig. 3A–C). The response of coarse root biomass was not related to aridity, soil N content or MAT (Fig. 3D–F), while the magnitude of eCO_2_ effects on total root biomass decreased with increasing aridity and MAT (Fig. 3H,I), but was not related to soil N content (Fig. 3G). We found a significant positive correlation between the size of eCO_2_ effects on fine root biomass and soil total C and a negative correlation between the size of eCO_2_ effects on fine root biomass and soil C:N ratio, although the correlation coefficients for these variables were weaker than for total soil N (Table 1). Soil pH, C:N ratio and soil C were not correlated with coarse or total root biomass responses to eCO_2_ (Table 1).

**Table 1:** Correlation coefficients (Pearson r) and p-value (in brackets) between soil pH, soil total C, Soil C:N ratio and mean annual precipitation (MAP) and the response ratios of total root biomass, coarse root biomass and fine root biomass. Bold number indicates significant p-values at 0.05. The sign indicates the direction of the slope. Aridity was calculated as [AI in this dataset – AI in each site] so increases in aridity represent places with low water availability.

	Total Root Biomass (ln RR)	Coarse Root Biomass (ln RR)	Fine Root Biomass (ln RR)
**Soil pH**	0.01 (0.67)	(−) 0.25 (0.11)	(−) 0.27 (0.09)
**Soil Total C**	0.07 (0.69)	(−) 0.19 (0.44)	**0.334 (0.04)**
**Soil C:N**	0.16 (0.35)	(−) 0.59 (0.65)	**(−) 0.35 (0.03)**
**MAP**	0.27 (0.10)	0.29 (0.33)	**(−) 0.32 (0.04)**

**Figure 4:**
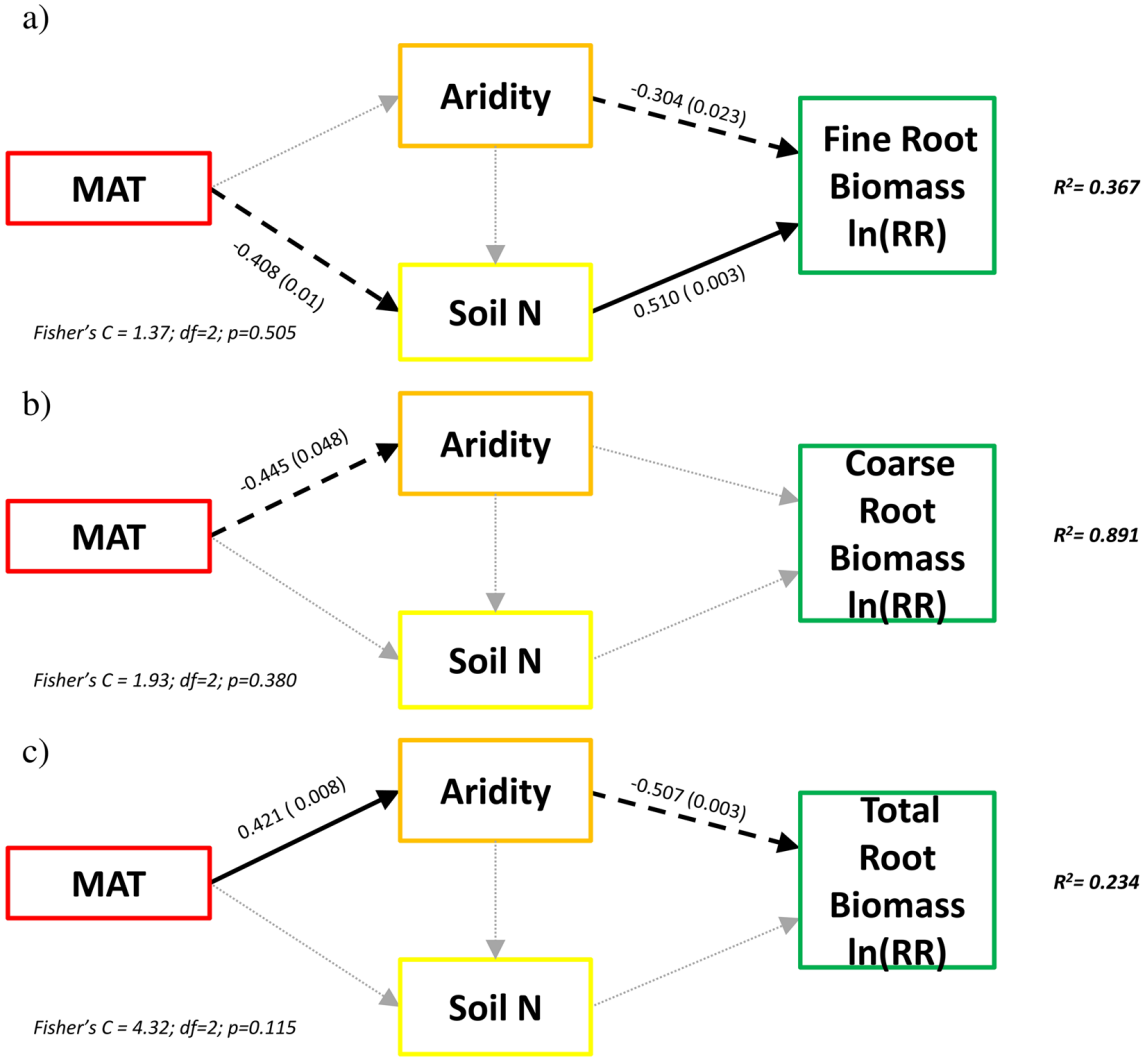
Effects of MAT, Aridity and soil N content on: (**a**) fine root biomass, (**b**) coarse root biomass and (**c**) total root biomass response to eCO_2_. Numbers adjacent to the arrows indicate the effect size of the relationship, while the p-values are shown in brackets. Continuous and dashed black arrows indicate positive and negative relationships, respectively, while grey arrows indicate non-significant relationships. R^2^ denotes the proportion of the model variance explained. Overall goodness-of-fit test is shown in the bottom of each figure. Aridity was calculated as [AI in this dataset – AI in each site] so increases in aridity represent places with low water availability.

Our SEMs explained 36%, 89% and 23% of the variance found in fine, coarse and total root biomass responses to eCO_2_, respectively (Fig. 4). Aridity had a direct, negative effect on the responses of fine and total roots to eCO_2_ (Fig. 4a,c). Moreover, there was a direct, positive relationship between soil total N and fine root responses to eCO_2_, but not for the total or coarse root pools (Fig. 4a–c). Effects of eCO_2_ on coarse roots were also unaffected by site aridity (Fig. 4b). Goodness of fit of all SEMs was examined using Fisher’s C tests (Fig. 4).

## Discussion

Our results provide evidence that enhancement of root biomass by eCO_2_ is lower at more arid sites, and greater where soil N content is high. Predicted increases in aridity^30^ may, therefore, limit future biomass responses of both fine and total roots as CO_2_ concentrations rise. Similarly, expected increases in N deposition^31^ and associated increases in soil N content may enhance fine root responses to eCO_2_, although effects on long-lived (coarse) root biomass are less clear. The data presented here summarize the responses of functionally different root compartments to field-based CO_2_ enrichment in a range of globally dominant ecosystem types (forests, shrublands and grasslands) with contrasting soil conditions and, therefore, have important implications for the understanding of root responses to eCO_2_ under changing environments.

The more limited stimulation of fine root biomass to eCO_2_ with increasing aridity may reflect a reduced need to allocate biomass to fine roots where concurrent gains in water use efficiency (WUE) occur. The magnitude of increased WUE under eCO_2_ has been predicted to be greater in arid-lands, which could explain the trend observed in our analysis^32^. It has been argued that eCO_2_ effects on arid-land vegetation tend to reduce the time plants are in water limitation, which prevents over-investment in fine roots^33^. This, in turn, may promote an increase in aboveground biomass at the expense of fine root biomass^32^. However, the lack of fine root biomass response to eCO_2_ under dry conditions does not necessarily imply a lack of eCO_2_ effect on fine root functional efficiency, since water and nutrient uptake may be more strongly related to other traits such as surface area, length or branching intensity^34,35^, rather than just biomass. Although there are relatively few data on root morphological responses to eCO_2_ under field conditions, those studies that have looked at this have reported shifts in these traits towards more acquisitive morphology (e.g. thinner, longer and more branched fine root compartments^5,6,12^). Our literature search and selection criteria did not, however, provide enough data from independent published studies to analyse this using SEM. Further understanding of the role of eCO_2_ in driving the responses of plant roots may be improved by including such functional variables.

Previous studies also reported contrasting fine root responses to eCO_2_ between wet and dry years within the same experiment; those in arid environments have reported a lack of eCO_2_ effects during dry periods^36,37^, while studies carried out in moderately or temporarily water-limited ecosystems show a greater relative increase in fine root investment in dry periods^38^. Taken together, our results and those of earlier studies suggest that eCO_2_ effects on fine root biomass differ between mesic and arid environments and, therefore, might be particularly influenced by plant life history and the environmental conditions experienced during plant development.

The decreasing (but still positive) response of the total root pool to eCO_2_ as aridity increased supports our second hypothesis, but challenges the general idea that plant biomass responses are greater (in relative terms) in water-limited ecosystems exposed to eCO_2_^22,39^. There is, however, some empirical support for our observation from field-based CO_2_ and water manipulation experiments^23,24^. The general hypothesis that coarse and/or total root growth under eCO_2_ will be higher under water-limited conditions is usually based on (i) the understanding that WUE typically increases, which may lead to higher soil water content^17,40,41^ and thus, overall growth; and (ii) the idea that a shift towards relatively greater belowground limitation will increase allocation of new biomass to roots. However, CO_2_-associated increases in soil water content tend to be relatively small and often restricted to short periods of time in arid ecosystems^42^ and may, therefore, be insufficient to sustain additional growth of long-lived plant biomass such as coarse/total roots in response to increases in aridity^39^. Furthermore, productivity responses of stress-tolerant species to eCO_2_ have been suggested to be lower than for those species associated with more mesic ecosystems^43,44^, which could also explain the smaller root responses to eCO_2_ in more water-limited ecosystems. A possible explanation for the lack of larger positive results in arid environments is that a minimum level of soil water is needed for plants to respond positively to eCO_2_ under water shortage^22^. Therefore, the previously observed greater positive effects of eCO_2_ on plant growth in dry periods may be restricted to mesic environments.

As we predicted, effects of eCO_2_ on fine roots increased as soil N increased. This is in agreement with previous findings that CO_2_ effects on fine root biomass were larger in N-rich forests compared to N-poor forests^21^. Given that soil N content mainly represents the organically-bound N pool (i.e. available for plants and microbes in the long-term), the authors argued that a higher biomass of active fine roots – and associated exudates - may be needed to increase N mobilization under higher rates of photosynthetic C assimilation. The mechanism underlying the smaller CO_2_ response of fine roots under N limitation may also be related to economic trade-offs between C fixation and N acquisition^33^. The carbon invested in root construction must be fixed from the atmosphere using an N-rich enzyme (i.e. Rubisco). Thus, increased investment of assimilates in fine root material under eCO_2_ pays for itself in terms of N uptake in N-rich ecosystems, while in N-poor ecosystems, the high C costs of fine root biomass construction and maintenance may restrict plant investment in fine roots.

The response of long-lived plant biomass such as coarse roots to eCO_2_ has typically been reported to be greater under higher N conditions in both grasslands and forests^9,20,45^. Surprisingly, we observed no effect of soil N content on coarse and total root biomass responses to eCO_2_. Several experimental and meta-analytical studies have demonstrated the context-dependent role of mineral N additions on belowground response to eCO_2_^6,11,45^. However, none of these studies have undertaken a quantitative evaluation of the role of soil N content (as opposed to just extractable N) in belowground biomass responses to eCO_2_. Other studies^46^ have shown a significant correspondence between increased rates of N mineralization and root biomass responses to eCO_2_ under contrasting soil N availabilities. Previous meta-analysis^21^ reported that the direction of eCO_2_ effects of fine root production differed when comparing N-fertilization experiments and N fertility gradients. For instance, fine root production response to eCO_2_ was lower in N-fertilized plots compared to unfertilized plots exposed to eCO_2_, but larger in soils with greater soil N content compared to those with lower N contents^21^. Under eCO_2_ conditions, plants may increase N uptake via priming of SOM decomposition^47^, and from exploration of deeper soil horizons^48^. Thus, increased C-cost of maintenance of higher fine root biomass and their activity in N-rich ecosystems (Fig. 3C) may preclude C investment to growth of long-lived coarse root tissues^18^. In addition, potential stoichiometric restrictions of other growth-limiting nutrients such as phosphorus may also limit the response of long-lived biomass to eCO_2_ despite increased N availability^49^. Other confounding factors such as the duration of CO_2_ treatment, plant age and the developmental stage of the study site may also help explain the lack of differences in the relative response of coarse/total root biomass to eCO_2_ along the N gradient.

Root responses to eCO_2_ may differ between ecosystem types (e.g., forests vs. grasslands), where fine roots can represent from as little as 10% (in some forests) to more than 70% (in some grasslands) of the standing root biomass^4,5^. Previous meta-analyses have reported similar (not statistically different) responses of fine and total root biomass to eCO_2_ (i.e. relative responses) between forest and grasslands^11,12^, supporting the approach that we adopted in this study. However, it is worth mentioning that the diameter-based classification of root types does not accurately account for functional differences between absorptive and transport/anchorage roots^50^, and thus, overestimates the percentage of the standing root biomass represented by absorptive roots, particularly in grasslands. An order-based classification could be more appropriate to capture functions (absorption, transport and anchorage) and dynamics (mortality and turnover) of roots under eCO_2_, particularly in trees, where root branching intensity tends to be higher and is also related to mycorrhizal colonization^51^. However, an order-based classification has rarely been carried out in field eCO_2_ experiments, which precluded this type of analysis in our dataset.

By comparing 24 field experiments at a global scale we have demonstrated that root responses to eCO_2_ appear to be constrained by high aridity and low total soil N. These findings may help inform ecosystem and Earth system model predictions of plant and ecosystem-scale responses to global change, particularly in the context of enhanced N deposition^31^ and the global expansion of arid ecosystems^30^. One goal of these models is to simulate feedbacks among ecosystem components and attributes (vegetation, microbes and resources) to predict ecosystem functions for contrasting biomes^52^. In such efforts, root pools are generally represented as fixed parameters (e.g. CLM4.5 and CABLE), which limits the ability of models to estimate, for example, the land C sink. Our results suggest that, although eCO_2_ tends to increase belowground biomass, intrinsic ecosystem properties (e.g. soil fertility) and climatic conditions significantly regulate the magnitude of such responses. Carbon, water and nutrient fluxes greatly depend on the biomass of different root components (i.e. fine vs coarse). Therefore, accurate model representation of the variability in responses of the different root fractions to eCO_2_ may improve model-based predictions of ecosystem functioning in response to changing environmental conditions. Predicted increases in atmospheric CO_2_ concentrations and N deposition may ultimately lead to ecosystems with higher fine root biomass. In contrast, however, the predicted expansion of arid-lands in a CO_2_-enriched world may result in lower-than-expected increases in belowground biomass.

## Material and Methods

### Data collection and extraction

We collected published data from the literature on the responses of root biomass to eCO_2_ and combined this information with selected climatic (mean annual temperature (MAT), mean annual precipitation (MAP) and aridity index [AI]), geographic (latitude and longitude) and edaphic (pH, organic matter content) data obtained for each study site. We used the meta-analysis published by Nie *et al*. (2013) as our starting point, but updated the database by searching for relevant studies (Open Top Chamber [OTC] and Free Air CO_2_ Enrichment [FACE] experiments) published between January 2012 and December 2015 using ISI WEB OF KNOWLEDGE^®^. We used the keywords “fine root biomass”, “fine root production” and “belowground productivity”, combined with “CO_2_”, “CO_2_ fumigation”, “FACE experiment”, “open top chamber” and “global change”. The resulting database led to 315 new articles that, along with the studies included by Nie *et al*. (2013), were then examined. Studies were only included in our analysis if they:

1. Reported results of experiments conducted under field conditions. Studies carried out in a greenhouse, growth chamber or using pots were not included.
2. Evaluated the effect of CO_2_ treatment on root biomass and production (fine, coarse and/or total). If the experiment was conducted with multiple species, each species was considered as a separate study case. If a published study reported results from multiple soil depths, root biomass results were averaged for the entire soil profile. Although many previous ecological meta-analyses only use results from the last time point of the experiment^53,54^, it can be argued that, for studies running for long periods (i.e. more than three years), the last point may not be representative of the whole experiment. Therefore, we ran statistical analyses with results averaged from multiple time points across the whole experiment. Given that root biomass responses to eCO_2_ may decrease from short- to longer periods of treatment^13,55^ due to more rapid root closure (defined as an equilibrium between production and mortality) in CO_2_-fumigated plots, when available, we included three time points for those experiments that had been running for more than three years (e.g. Duke FACE, ORNL FACE. In those studies where other treatments (e.g. watering or fertilization) were also applied, we only considered responses to CO_2_ under ambient conditions for these factors. Given that the main focus of our study is to identify major environmental predictors of the responses of roots to eCO_2_ in unmanaged ecosystems, the inclusion of other treatments would have obscured the relationships between root responses to eCO_2_ and climatic or fertility predictors.
3. Application of these criteria resulted in sufficient data to calculate the CO_2_ logarithm response ratio (ln RR), obtained as^27^:

1 $$\mathrm{ln}\,{\rm{RR}}=\,\mathrm{ln}({\rm{Xt}}/{\rm{Xc}})$$

where Xt and Xc are the means of the treatment and control, respectively. When the results were presented graphically, data were extracted using Datathief (www.datathief.org).

For each experiment, the following information was obtained: (1) duration of experimental treatment, (2) soil properties (pH, total C, total N, CN ratio, texture) and (3) ecosystem type (cropland, grassland, shrubland or forest). When such information was not available, authors were contacted to obtain the original data. Mean annual temperature (MAT), mean annual precipitation (MAP) and aridity index (AI; mean annual precipitation/potential evapotranspiration) for each study site were obtained from the WorldClim database^56^, which provides average climatic values for the period 1950–2000. To improve interpretation, aridity was presented as [maximum value of AI in this dataset – AI in each site] (see^57^ for a similar approach). Thus, aridity - which is negatively related to AI (r = −1.0; *P* < 0.001) - represents a metric of water scarcity, instead of a metric of water availability.

### Statistical analyses

We used Structural Equation Modelling^58^ (SEM) to assess the direct and indirect effects of multiple climatic and soil properties on the responses of root biomass to eCO_2_. Moreover, the use of SEM allowed us to partition the effects that one predictor variable may have on a response variable, and to estimate the strengths of these multiple effects. However, due to the high number of environmental drivers potentially affecting root responses to eCO_2_, we first used Pearson correlations to explore the association between the effect sizes (ln RR) of fine, coarse and total root biomass and potential explanatory variables (climatic, geographic and edaphic conditions). We only included in our SEM those factors that were shown to be correlated with root responses. Then we established an *a priori* model (Fig. 2) based on previous knowledge and identified correlations. Since studies along climatic and fertility gradients suggest that temperature, water and N content are the main factors controlling root biomass differences between ecosystems^3^, our *a priori* model incorporated these variables^59^. In accordance with the literature, MAT, aridity and soil total N showed the strongest relationships in the correlation analysis (Table 1; Fig. 3) and so were included in our models as predictor variables. Given the limited number of independent studies that met our selection criteria or provided enough data, we included data from different species within a site and from different publications within the same experiment (for those experiments running for more than three years) in order to meet the data requirements of SEM.

This approach resulted in a hierarchical data structure (i.e. multiple measurements of fine, coarse or total root biomass belonging to different species and time points within experimental sites), which precluded the use of the maximum likelihood method of standard SEM analysis^58^. We therefore, adopted Shipley’s d-sep method for model evaluation. This approach avoids pseudoreplication by constructing SEM paths as a set of hierarchical linear mixed effects models^60,61^ and has been suggested to have higher statistical power in studies with small sample size^59,62^. We fitted linear mixed models for fine, coarse and total root biomass incorporating MAT, aridity, soil total N content as fixed effects, and time and species nested within experimental site as random factors. Linear mixed models were fitted using the “lme” function in the “nlme” package^62^ and model assumptions were verified by inspecting residuals versus fitted values and quantile–quantile plots. Directional separation analyses were carried out with the “piecewiseSEM” package in R^62^. Overall goodness-of-fit of the models was tested using Fisher’s C statistic^60,61^. Non-significant P-values associated with goodness-of fit tests indicate acceptable model fit. Data were transformed when needed to improve linearity in the correlation analysis and SEM models. All statistical analyses were performed using experiment-averaged results.

Given the relatively small number of studies that complied with our selection criteria when compared with other meta-analyses^11,12^ (see Results), we conducted a standard meta-analysis to elucidate whether our dataset is representative of a larger set of studies. To do so, we calculated cumulative effect sizes for fine, coarse and total root biomass, along with heterogeneity and publication bias assessments (a detailed methodology and results are included in Supplementary Information). Our results are in accordance with previous findings; eCO_2_ increased fine, coarse and total root biomass by 15, 24 and 23% respectively. In all cases, heterogeneity tests were significant, suggesting that other factors (e.g. climate and soil properties) should be evaluated. In addition, we did not detect publication bias in our dataset, which suggests that our data can be considered as to be a good representation of a larger number of experiments. The finding that only 24 studies complied with our selection criteria (see results) reflects the fact that a large proportion of published studies involving CO_2_ manipulation had either not been conducted under strictly natural conditions, or did not include an adequate description of the ecosystem’s properties. We call upon the authors of future studies to fully characterize ecosystem properties, so that they can be incorporated into potential meta-analyses.

## Electronic supplementary material

Supplementary Information

Coarse root

Fine root

Total root
