# Supplementary material for: Effects of elevated CO2 on fine root biomass are reduced by aridity but enhanced by soil nitrogen: A global assessment
Source: Sci Rep. 2017 Nov 10;7:15355. doi: 10.1038/s41598-017-15728-4 (PMC5681551; doi:10.1038/s41598-017-15728-4)
Supplement: Supplementary file 1 — Supplementary Information [file 41598_2017_15728_MOESM1_ESM.doc]

**Supplementary Information**

**Effects of elevated CO_2_ on fine root biomass are reduced by aridity but enhanced by soil nitrogen: A global assessment.**

Juan Piñeiro*^1^, Raúl Ochoa-Hueso^1^, Manuel Delgado-Baquerizo^1^, Silvan Dobrick^1^, Peter B. Reich^1^,^2^, Elise Pendall^1^ and Sally A. Power^1^.

^1^Hawkesbury Institute for the Environment, Western Sydney University, Locked Bag 1797, Penrith, New South Wales, 2751, Australia.

^2^Department of Forest Resources, University of Minnesota, St. Paul, Minnesota, USA

1. Meta-analysis: Statistical procedure

Independent meta-analyses were carried out for each response variable (i.e. fine root biomass (FRB), coarse root biomass (CRB) and total root biomass (TRB)). To estimate the cumulative treatment effect (TE), we used the natural logarithm of the response ratio (ln RR), (Hedges et al. (1999):

ln RR=ln(Xt/Xc) (1)

where Xt and Xc are the means of the treatment and control, respectively. The variance of this ratio is calculated as follows:

(2)

where Xt and Xc are the means of the treatment and control, respectively, Nt and Nc are the number of replicates of the treatment and the control, respectively, and Dt and Dc are the standard deviations of the treatment and the control, respectively. We extracted means (and standard deviations) from each study as explained in the main text. Briefly, when a published study reported results from multiple time points, root biomass results (i.e. means ±SD or SE) from each time point were summed and divided by the number of time points. Similarly, when a published study reported results from multiple soil depths, results (means ±SD or SE) from each depth were summed and divided by the number of depths. We used values averaged across all reported depths because this is a more conservative approach that is potentially less sensitive to differences in distribution of root biomass at different time points/depths.

In cases where all the information could be retrieved (mean, n and SD; see Appendix 1) we used a random effects model to calculate the treatment effect (TE), which assumes that the studies included are a random selection from a larger population of studies. Random effects meta-analyses are usually preferred because the fixed-effects model assumption that all observed variation is due to sampling error is very difficult to meet when using a broad range of studies (Rosenberg et al., 2000). The total heterogeneity, Q_T_, was also calculated; this is simply Cochran’s Q-test (Cochran, 1954), which tests whether the variability in the observed effect sizes is larger than would be expected based on sampling variability alone. A significant value of this statistic indicates that the variance among effect sizes is greater than expected by sampling error (Rosenberg et al., 2000), suggesting that other factors should be evaluated.

We tested for publication bias by checking normal quantile plots and funnel plot asymmetry (Rosenberg et al., 2000). Funnel plot asymmetry was tested using rank correlation tests as described by Begg and Mazumdar (1994), which examines whether the observed outcomes and the corresponding sampling variances are correlated. A significant value would indicate that the funnel plot is asymmetric, suggesting publication bias. With publication bias, normal quantile plots are non-linear or contain unusual gaps. All statistical meta-analysis were performed in R with the “metafor” package.

2. Meta-analysis: Results

Our results are in accordance with previous findings (e.g. de Graaff et al., 2006, Dieleman et al., 2010, Sillen & Dieleman 2012); eCO_2_ increased fine, coarse and total root biomass by 15, 24 and 23% respectively (Fig. S1). In all cases, heterogeneity tests were significant (Table S1), suggesting that other factors (e.g. climate and soil properties) should be evaluated. In addition, we did not detect publication bias in any of our datasets (Fig.S2), implying that our data can be considered as a good representation of a larger number of experiments.

**Table S1.** Summary of total heterogeneity analysis and publication bias

Table 1


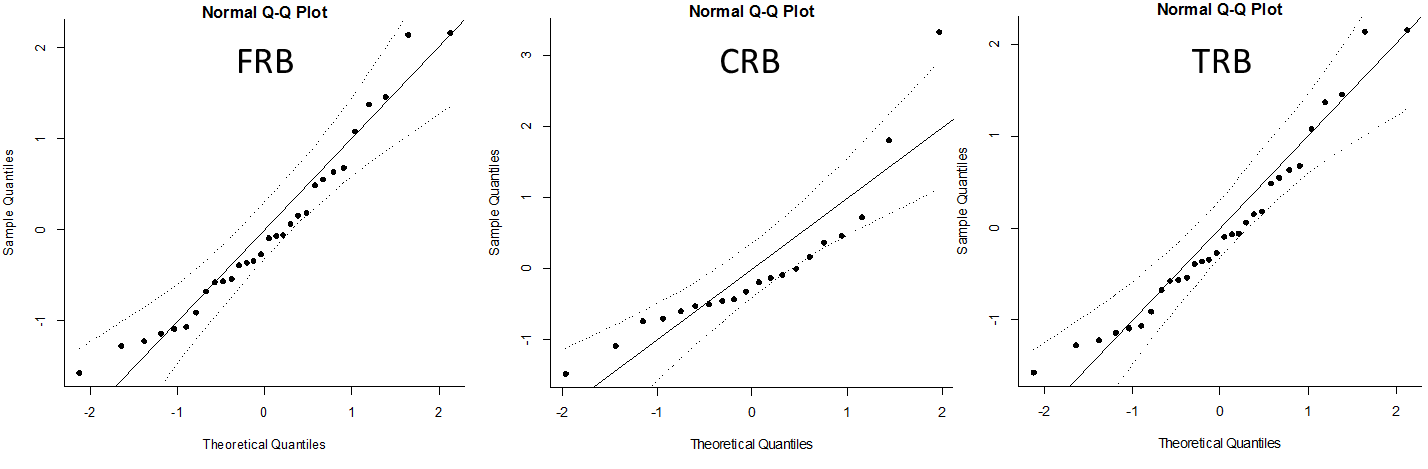


**Figure S2.** Normal quantile plot of fine root biomass (FRB), coarse root biomass (CRB) and total root biomass (TRB) data

**References:**

Begg, C. B., & Mazumdar, M. (1994). Operating characteristics of a rank correlation test for

publication bias. Biometrics, 50, 1088–1101.

Borenstein, M., Hedges, L. V., Higgins, J., & Rothstein, H. R. (2009). References (pp. 409-414). John Wiley & Sons, Ltd.

Cochran, W. G. (1954). Some methods for strengthening the common χ2 tests. Biometrics, 10, 417–451.

De Graaff, M.-A., Van Groenigen, K.-J., Six, J., Hungate, B. & Van Kessel, C. Interactions between plant growth and soil nutrient cycling under elevated CO2: a meta-analysis. Global Change Biology 12, 2077-2091 (2006).

Dieleman, W. I. J. et al. Soil [N] modulates soil C cycling in CO2-fumigated tree stands: a meta-analysis. Plant, Cell & Environment 33, 2001-2011 (2010).

Rosenberg, M.S., Adams, D.C., Gurevitch, J., 2000. Metawin: Statistical Software for Meta-Analysis, Version 2. Sinauer Associates, Sunderlan.

Sillen, W. M. A. & Dieleman, W. I. J. Effects of elevated CO2 and N fertilization on plant and soil carbon pools of managed grasslands: a meta-analysis. Biogeosciences 9, 2247-2258 (2012).
